# Toward an understanding of the DNA replication initiation in bacteria

**DOI:** 10.3389/fmicb.2023.1328842

**Published:** 2024-01-05

**Authors:** Katarzyna Wegrzyn, Igor Konieczny

**Affiliations:** Intercollegiate Faculty of Biotechnology of University of Gdansk and Medical University of Gdansk, University of Gdansk, Gdansk, Poland

**Keywords:** DnaA, Rep proteins, DNA unwinding element, origin opening, two-state model, loop-back model

## Abstract

Although the mechanism of DNA replication initiation has been investigated for over 50 years, many important discoveries have been made related to this process in recent years. In this mini-review, we discuss the current state of knowledge concerning the structure of the origin region in bacterial chromosomes and plasmids, recently discovered motifs recognized by replication initiator proteins, and proposed in the literature models describing initial origin opening. We review structures of nucleoprotein complexes formed by replication initiators at chromosomal and plasmid replication origins and discuss their functional implications. We also discuss future research challenges in this field.

## 1 Introduction

DNA replication in bacteria has been studied for decades since the concept of replicon was proposed (Jacob and Brenner, 1963) and the gene for the DnaA protein (Emmerson and Kohiyama, 1971) as well as the origin of *E. coli* chromosome region (*oriC*) (Yasuda and Hirota, 1977) were identified. The last 15 years have been a time of intensive exploration of the structure of replication initiation complex and the mechanism of action of replication initiation proteins. Diverse research models (bacterial chromosomes, plasmids, and phages) have been utilized to investigate the DNA replication initiation and the obtained results bring us closer to putting together the puzzles regarding the mechanism of this process. The simple *oriC* structure containing five canonical-binding sites for DnaA (R-type DnaA-boxes) (Fuller et al., 1984) has been expanded with identified additional binding sites for this replication initiator (Kawakami et al., 2005; Miller et al., 2009; Rozgaja et al., 2011). New activities such as binding of single-stranded DNA (ssDNA) within the DNA unwinding element (DUE) region by bacterial replication initiators (DnaA and RctB) were discovered (Ozaki et al., 2008; Duderstadt et al., 2011; Chatterjee et al., 2020). The formation of nucleoprotein complexes with ssDNA DUE was also identified for plasmid replication initiators (Rep proteins) (Wegrzyn et al., 2014) and new structures of replication initiators were published (Orlova et al., 2017; Wegrzyn et al., 2021). Although many new methods were utilized over these years and the knowledge on DNA replication has been broadened, the detailed mechanism of DNA replication initiation is still discussed and there are still many questions that require to be answered.

## 2 Structure of replication origin regions

**Figure 1:**
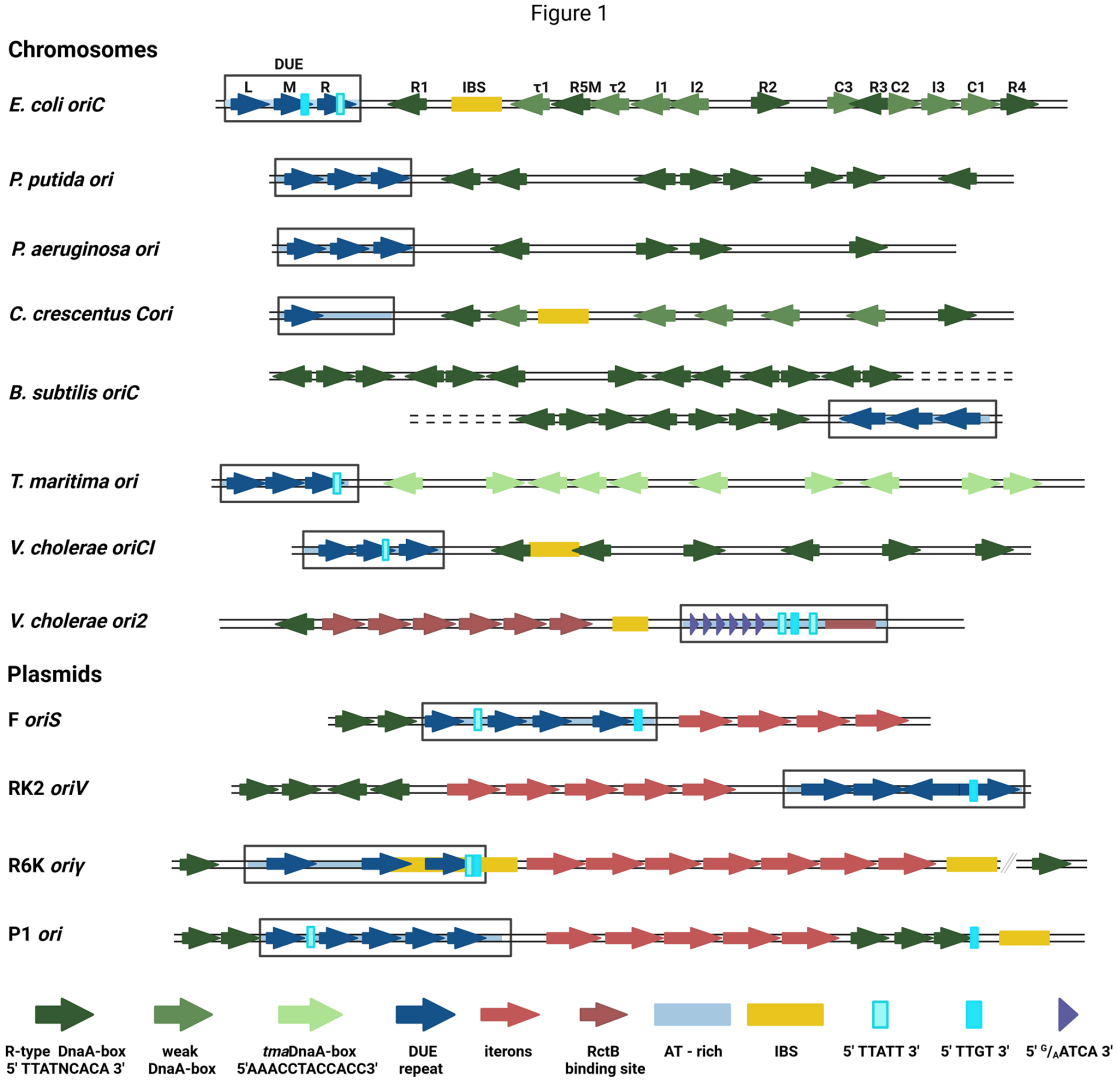
The structures of selected bacterial chromosomes’ and plasmids’ origin regions. In the schemes, the motifs and sequences are marked as colored arrows and rectangles: R-type DnaA-boxes (dark green arrow), weak-DnaA-boxes (light green arrow), *tma*DnaA-boxes (very light green arrows), AT-rich region (light blue shading), repeat in DUE region (dark blue arrow), DUE (rectangle), Integration Host Factor binding site (IBS) (yellow rectangle), Rep proteins binding sites (iterons) (red arrows), RctB binding sites (dark red arrows and red rectangle), *E.coli* DnaA binding sites in DUE 5′TTATT3′ (light cyan rectangle) and 5′TTGT3′ (cyan rectangle), *V. cholerae* ori2 repeats in DUE 5′^G^/_A_ATCA3′ (short purple arrow). In *E. coli oriC*, the names of particular motifs are written above the scheme.

DNA replication initiation in bacteria starts with replication initiation proteins binding with the specific motifs within a well-defined origin region (Wolanski et al., 2014; Leonard and Grimwade, 2015), typically near the DUE region, where double-stranded DNA (dsDNA) melts (Rajewska et al., 2012). In bacteria, chromosome replication relies on the activity of the four-domain DnaA protein (Leonard et al., 2019). Domain I is responsible for DnaA dimerization and interaction with other proteins (DnaB and DiaA), domain II is a linker, domain III is a nucleotide- and ssDNA-binding domain, and domain IV binds dsDNA (Hansen and Atlung, 2018). Bacterial chromosomes have a varied number of binding sites for DnaA (DnaA-boxes) at replication origin. In most analyzed bacterial genomes, the sequence of DnaA-boxes aligns with the *E. coli* R-type consensus (5′TTATNCACA3′), with a possible single mismatch, except *Thermotoga maritima* where they significantly differ (5′AAACCTACCACC3′) (Wolanski et al., 2014). Currently, in *E. coli oriC* there are defined 12 DnaA-boxes bound by DnaA with different affinity (Rozgaja et al., 2011) (Figure 1). The sequence of non-R DnaA-boxes is more degenerate compared with the consensus of R-type (Leonard et al., 2019). The thirteenth DnaA-box, R3 is often omitted since it is overlapped with C2 and C3 DnaA-boxes. In dimethyl sulfate (DMS) footprinting experiments, only minor changes in modification pattern were seen in the R3 region (Rozgaja et al., 2011). Five DnaA-boxes (R1, R5M, τ2, I1, and I2) from the left part of the DnaA oligomerization region (left-DOR) were sufficient for the unwinding of DUE (Ozaki and Katayama, 2012). The DnaA binding to τ1 site, also within the Left-DOR, was shown in DNaseI footprinting (Kawakami et al., 2005); however, it was not detected in the DMS footprinting assay on the supercoiled template and this interaction seems not to occur when integration host factor (IHF) binds to its binding site (IBS) (Rozgaja et al., 2011). The remaining DnaA-boxes (except R3) are directed opposite those in Left-DOR. A similar orientation of DnaA-boxes was also identified in *Vibrio cholerae* chromosome I (oriC1), in *Bacillus subtilis* and *Pseudomonas aeruginosa* (Wolanski et al., 2014) (Figure 1). In *Caulobacter crescentus,* two oppositely directed DnaA-boxes (named G-boxes) (Shaheen et al., 2009) and additional five weak-binding sites (W-boxes) (Taylor et al., 2011) were identified. Weak binding sites for DnaA have not yet been identified in other bacteria. In addition to bacterial chromosomes, DnaA-boxes are also identified in plasmid origins, although DnaA is not the main plasmid replication initiator but assists a plasmid-encoded protein (Rep) in DNA replication initiation (Konieczny et al., 1997). Plasmid-origin DnaA-boxes sequences share similarities with the *E. coli* R-type box, and their number varies depending on the plasmid (Rajewska et al., 2012).

The detailed analysis of the sequence required for bacterial chromosome replication initiation revealed other motifs recognized by DnaA. In *B. subtilis* (Richardson et al., 2016) and other bacteria (Jaworski et al., 2021; Pelliciari et al., 2021), the three-nucleotide motif named DnaA-trio (3′^G^/_A_AT5′), located close to the DnaA-box nearest to DUE and repeated a few times (Richardson et al., 2016), was identified. In *E. coli oriC*, the DnaA-trio overlays with the 5′TTATT3′ sequence, bound as ssDNA by DnaA (Katayama et al., 2017) (Figure 1). Another ssDNA sequence required for *E. coli* DnaA interaction with ssDNA is 5′TTGT3′ (Ozaki et al., 2008) (Figure 1). Both motifs partially overlap with the 13-nucleotide repeats (R and M) distinguished within DUE. In *oriC* DUE, there is a third 13-mer named L, not essential *in vivo* (Kowalski and Eddy, 1989). The 13-mers were also identified in the origins of other bacteria, e.g., *P. aeruginosa*, and *V. cholerae* (Rajewska et al., 2012), although their sequences differ between the organisms. Repeated sequences were also found in the origins of phage λ and bacterial plasmids, although they vary in length, spacing, and sequences between replicons (Rajewska et al., 2012). In one of the best-characterized plasmid origins, *oriV* from broad-host-range plasmid RK2, four 13-mers were identified in DUE (Konieczny et al., 1997) (Figure 1). All these repeats are required for plasmid DNA replication and point mutations in their sequence inhibit plasmid replication *in vivo* and *in vitro* (Kowalczyk et al., 2005; Rajewska et al., 2008; Wegrzyn et al., 2014). The strand-specific interaction between plasmid-encoded Rep and ssDNA DUE was shown (Wegrzyn et al., 2014) and recent structural data on RepE protein of plasmid F complexed with ssDNA showed that thymidine residues in DUE are important for this interaction (Wegrzyn et al., 2023). Repeated sequences present in many DUE are rich in thymidine residues (Rajewska et al., 2012), so these residues might be important also for the interaction of other replication initiators. The unique motifs recognized by the initiator protein are also possible like in the second chromosome of *V. cholerae* (Chr2) ori2 (5′^G^/_A_ATCA3′) (Chatterjee et al., 2020). Data published so far do not indicate DnaA interaction with ssDNA DUE of plasmids; however, in the plasmid origins, the *E. coli oriC* motifs (5′TTATT3′ and 5′TTGT3′) are present (Figure 1). Therefore, the host initiator may interact with these sequences.

Replication initiation of iteron plasmids (e.g., RK2, F, or R6K), containing within origin direct repeats named iterons, depends on plasmid-encoded Rep protein, consists of at least two winged-helix domains (WH) (Kim et al., 2020). Reps first recognize and bind to dsDNA origin’s iterons, varying in length and spacing depending on a plasmid (Rajewska et al., 2012; Wegrzyn et al., 2016). Efficient binding of iterons requires their precise number, location, and sequence. Point mutations in the sequence of iterons or their spacers (McEachern et al., 1985; Brendler et al., 1997) and insertion of a half-helical turn between them (Bowers et al., 2007) affect Rep binding and replication activity. Recent data showed that the sequence-specific binding of iterons within *oriV* requires all Rep protein domains to interact with DNA (Wegrzyn et al., 2021). Similar to low-affinity DnaA-boxes within *oriC*, a prediction of potential weak binding sites for Rep proteins was made (Bowers et al., 2007); however, to date, there is no evidence of such sequences in plasmid origins. There is also no evidence that Rep proteins form a filament. However, insertions between iterons or reduction of their number affect Rep binding most likely by influencing the cooperativity (Perri et al., 1991; Perri and Helinski, 1993). Protein–protein interactions were also shown for the bacteriophage λ replication initiator (O protein), which binds to four iterons in *oriλ*. The sharp bending of DNA after O protein binding was shown and the formation of O-some structure, similar to early models of DnaA-*oriC* complexes (Bramhill and Kornberg, 1988), was proposed (Schnos et al., 1989). Unfortunately, the structure of O protein bound to iterons has not yet been solved, although some attempts were made (Struble et al., 2007).

## 3 Models for initial origin opening

### 3.1 Two-state model of the replication initiation complex

**Figure 2:**
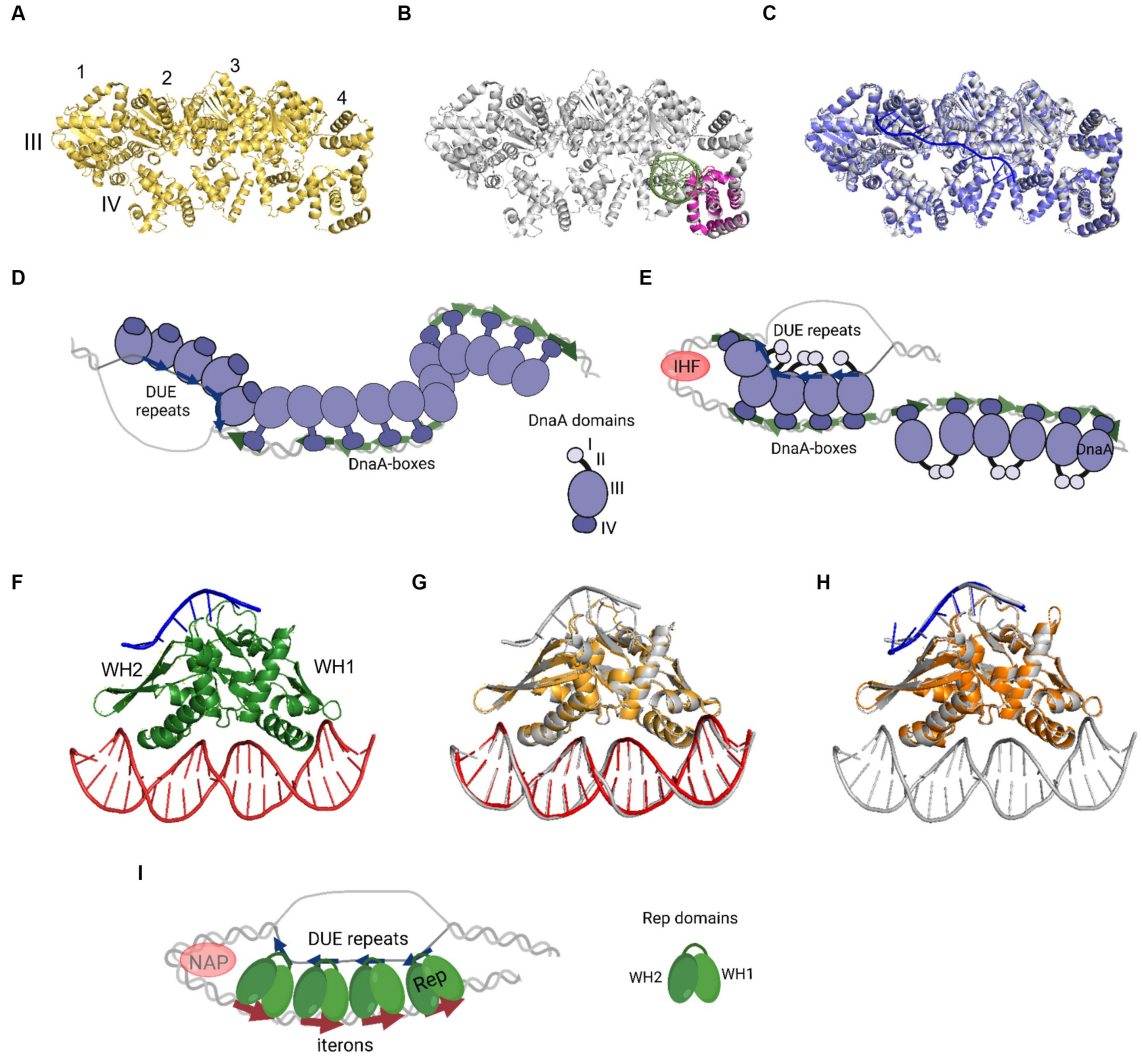
The nucleoprotein complexes formed by replication initiator proteins bacterial DnaA and plasmid Rep proteins. **(A)** The oligomeric structure of four protomers of domains III and IV of *Aa*DnaA PDB: 2HCB (yellow). Each protomer is numbered and domains III and IV are indicated. **(B)** Alignment of PDB: 2HCB structure of *Aa*DnaA (gray) and PDB: 1J1V structure of IV domain of *Ec*DnaA protein (pink) in the complex with dsDNA (light green). **(C)** Alignment of PDB: 2HCB structure of *Aa*DnaA (gray) and PDB: 3R8F structure of domains III and IV of *Aa*DnaA (light and dark purple) in a complex with ssDNA (blue). **(D)** The two-state model of bacterial origin opening based on Ekundayo and Bleichert (2019). **(E)** The loop-back model of bacterial origin opening based on Katayama et al. (2017). **(F)** The PDB: 8AAN structure of RepE protein (green) in a complex with dsDNA (red) and ssDNA (blue). The WH1 and WH2 domains are indicated. **(G)** The alignment of PDB: 8AAN tripartite RepE nucleoprotein complex structure (gray) and PDB: 1REP structure of RepE (light orange) in a complex with dsDNA (red). **(H)** The alignment of PDB: 8AAN tripartite RepE nucleoprotein complex structure (gray) and PDB: 8 AC8 structure of RepE (orange) in a complex with ssDNA (blue). **(I)** The loop-back model of plasmid origin opening based on Wegrzyn et al. (2023). **(D,E,I)** DnaA (purple), DnaA-boxes (green arrows), DUE repeats (blue arrows), Rep protein (green), iterons (red arrows), and NAP protein, e.g., IHF (light red) are presented. The alignment of the structures was done with PyMOL software (PyMOL Molecular Graphics System, version 2.4.0, Schrödinger, LLC; http://pymol.org/).

In 1993, Kornberg and co-workers presented electron microscope data showing that DnaA forms a compact structure in *oriC* (Crooke et al., 1993). The proximity of a multitude of DnaA-boxes enables the interaction of individual protein protomers, leading to filament formation (Figures 2A–E). However, the structural complexity arises from DnaA’s interaction with ssDNA DUE, and consideration of both binding to dsDNA DnaA-boxes and DnaA-trio or other motifs. One of the mechanisms by which the complex of DnaA in bacterial origin might be formed is called the “two-state model” (“DNA continuous filamentation model”) (Figure 2D). It assumes that DnaA, bound to DnaA-boxes, forms a filament and continues to ssDNA DUE. This is supported by structural data obtained for truncated *Aquifex aeolicus* DnaA (*Aa*DnaA) protein, showing a right-handed superhelix formed by specific protein–protein interactions (Figure 2A) (Erzberger et al., 2006). The *E. coli* DnaA’s domain IV in a complex with dsDNA was docked to this structure, showing how the DnaA filament on dsDNA might look (Duderstadt et al., 2010). The biochemical analysis of *Aa*DnaA and *Ec*DnaA protein variants with substitutions in arginine finger and the interface of interaction between adjacent domains III and IV support the importance of protein–protein interactions between DnaA molecules in filament formation on dsDNA (Duderstadt et al., 2010). The second crystal structure of truncated *Aa*DnaA reveals a spiral configuration of four protomers along the bound ssDNA (Ozaki et al., 2008; Duderstadt et al., 2011). The biochemical analysis of point mutants indicated that the domain III residues are important for DnaA–ssDNA DUE interaction (Ozaki et al., 2008; Duderstadt et al., 2010). In the two-state model, it is proposed that after DnaA binds to DnaA-boxes, additional DnaA protomers bind to DUE, increasing destabilization of the duplex within the AT-rich region. Next, after DUE melting, ssDNA can be sequestered by the stable association of DnaA via domain III. Dudersdat and co-workers demonstrated that DnaA can directly melt short DNA duplexes, supporting the proposed enhancement of DUE melting by DnaA bound in this region and the following sequestration of ssDNA (Duderstadt et al., 2011). The influence of formed DnaA filament on the unwinding of duplex *in vitro* was also shown in research conducted with the *B. subtilis* system (Richardson et al., 2019). The two-stage model can also be supported by similarities of this model to the origin opening mechanism by some viruses and eukaryotic organisms (Duderstadt and Berger, 2013). However, the available data do not explain how the initial recruitment of DnaA to double-stranded DUE could occur. Although there is data available showing the interaction of ATP-bound DnaA to 6-bp sequence, ATP-DnaA-boxes, within DUE (Speck and Messer, 2001), more evidence for this phenomenon is lacking. The second debatable issue is the protein structure within the oligomers. It is proposed that DnaA structure is different in the oligomer bound to DnaA-boxes when compared to the oligomer assembled on ssDNA DUE and it is more extended in the first one. This proposed structure results in efficient binding of both high- and low-affinity DnaA-boxes via DnaA domain VI (Duderstadt et al., 2010). In the DnaA–ssDNA complex, the structure is proposed to be more compact to enable the interaction between the adjacent protein protomers. The suggested conformational changes in DnaA domains allowed to explain DnaA binding to dsDNA, ssDNA, and interaction with adjacent protomers, but there is a lack of structural data supporting these assumptions. Aligning available crystal structures of *Aa*DnaA oligomers with and without ssDNA and of *Ec*DnaA domain IV-dsDNA complex reveals minimal structural differences (Figures 2A–C). If we consider the replication as a process that follows a similar mechanism in bacterial replicons, then the recently published structural data concerning plasmid Rep proteins, also do not support conformational changes of replication initiator during DNA replication initiation (Wegrzyn et al., 2023). No structural changes were observed for the RepE protein in complexes with dsDNA, ssDNA, and both of them (Figures 2F–H). If the two-state model is correct, then obtaining structural data of the DnaA protein oligomer in complex with dsDNA as well as bound to dsDNA DUE would strengthen it. It is also worth noticing that the two-state model does not address the arrangement of DnaA domain I, responsible for protein–protein interactions, within the nucleoprotein complex.

### 3.2 Loop-back model

The replication initiator oligomer formation is also proposed in the “loop-back model” (“ssDNA recruitment model”); however, in this model, it is assumed that DnaA initially binds to DnaA-boxes, and the same protein molecules then interact with the unwound ssDNA DUE (Ozaki and Katayama, 2012) (Figure 2E). The biochemical assays indicated the crucial role of DnaA binding to R1 and R5 DnaA-boxes for ssDNA recruitment (Ozaki and Katayama, 2012; Sakiyama et al., 2017). This model also proposes the interaction between domain III of adjacent DnaA protomers, considering, however, that DnaA bound to DnaA-box R2 does not interact via domain III with DnaA bound to I2 and C3 (Rozgaja et al., 2011; Shimizu et al., 2016). To support the cooperativity of binding, the interaction of domains I might occur (Katayama et al., 2017). Despite differences in motif organization within the origin of *T. maritima* (see Section 2), a similar model of binding and origin opening is proposed for this bacterium (Lu et al., 2023). Because the “loop-back model” assumes the definite bending of DNA, the assistance of nucleoid-associated proteins (NAPs) is proposed. In *E. coli oriC,* IHF binding to IBS, located between the R1 and R5M DnaA-boxes (Figure 1) might facilitate DnaA protomers to come closer together and their domains III closer to ssDNA DUE. Although the IBS is present only in some bacteria species, another NAP, the HU protein, is present more widely. It was shown that in *B. subtilis*, HU homolog (HBsu protein) is required for chromosome replication initiation (Karaboja and Wang, 2022), and in *E. coli*, despite sequence-unspecific binding, HU can replace the IHF during *oriC* unwinding *in vitro* and *in vivo* (Hwang and Kornberg, 1992; Ryan et al., 2002; Chodavarapu et al., 2008). Recent data demonstrated that, unlike IHF, HU binding and bending of *oriC* rely on prior R1 and R5M binding by DnaA due to the lack of sequence-specificity (Yoshida et al., 2023). The orientation of these two DnaA-boxes might facilitate the initial bending of origin by the R1/R5M-DnaA complex formation. That next can facilitate HU binding between these DnaA-boxes since HU has a higher affinity to bend DNA (Bonnefoy and Rouviere-Yaniv, 1991; Kamashev et al., 2017; Yoshida et al., 2023). The HU binding between two DnaA-boxes occupied by DnaA was also shown for *T. maritima* origin and the sequence analysis of bacterial origins often revealed the required distance (Koh et al., 2011) between the first and second DnaA-box adjacent to DUE for HU-induced sharp DNA bending (Lu et al., 2023; Yoshida et al., 2023). Although most of the research concerning looping of the DNA as a mechanism of origin opening is based on experiments with DnaA, there are also studies showing that other DNA replication initiators may follow the loop-back mechanism (Chatterjee et al., 2020; Wegrzyn et al., 2023). The analysis of ternary complex formation between replication initiators, either RctB of *V. choleare* or plasmid Rep proteins (TrfA and RepE), dsDNA and ssDNA showed that these proteins could interact with two types of DNA in a sequence-specific manner (Wegrzyn et al., 2014; Chatterjee et al., 2020; Wegrzyn et al., 2023). Although those experiments did not determine if one molecule of RctB or Rep accommodates both dsDNA and ssDNA, the recently published biochemical and structural data dispels these doubts (Wegrzyn et al., 2023). The *in vitro* replication assay and analysis of open complex formation indicates that Rep molecules have to be capable of binding both dsDNA and ssDNA to be active in plasmid DNA replication. The structure of RepE protein in a tripartite complex brings the solid data that one molecule of plasmid replication initiator can bind both ds DNA and ssDNA at the same time. Moreover, the orientation of iteron and DUE sequences in this structure is consistent with the looping assumptions in the proposed model (Figure 2I). Also, the involvement of NAP proteins seems plausible if we take into account the structure of the origin of many plasmids as well as *V. cholerae* Chr2 (Figure 1). In Chr2 ori2 (Chatterjee et al., 2020), the origin of P1 (Fekete et al., 2006), R6K (Lu et al., 1998) and pSC101 (Stenzel et al., 1987) plasmids the IBS can be distinguished. If there is no IBS identified, the action of other NAP, such as HU, was shown to be required for plasmid DNA replication (Ogura et al., 1990; Konieczny et al., 1997; Zzaman et al., 2004). There is enough space to bind HU between DUE and iterons in the sequence of these plasmid origins.

## 4 Perspectives

The recently obtained data shed new light on how the replication origin is opened, a critical step during DNA replication initiation. The looping-back mechanism is the most supported by experimental data and filaments formed by replication initiators seem to be essential for initiation complexes. Advances in structural techniques, such as cryo-EM microscopy, offer promise in elucidating the structure of a complete replication initiation complex comprising DnaA and/or Rep oligomers and nucleoid-associated proteins (NAPs). This initial complex recruits helicase and polymerases to the ssDNA generated by replication initiators. It must be highly dynamic since its formation regulates DNA replication initiation. It was demonstrated that DnaA protein phosphorylation and acetylation status affect its replication activity (Zhang et al., 2016; Lebkowski et al., 2020); however, how exactly it affects the initial complex formation and structure is yet unknown. Finally, the regulatory role of DnaA within the initial complex in controlling the replication of extrachromosomal replicons like plasmids is still unclear. These and other questions concerning the replication initiation process still wait to be answered.

## Author contributions

KW: Writing – original draft, Writing – review & editing. IK: Writing – review & editing.
